# Advancements in proteogenomics for preclinical targeted cancer therapy research

**DOI:** 10.52601/bpr.2024.240053

**Published:** 2025-02-28

**Authors:** Yuying Suo, Yuanli Song, Yuqiu Wang, Qian Liu, Henry Rodriguez, Hu Zhou

**Affiliations:** 1 Department of Analytical Chemistry, State Key Laboratory of Drug Research, Shanghai Institute of Materia Medica, Chinese Academy of Sciences, Shanghai 201203, China; 2 University of Chinese Academy of Sciences, Beijing 100049, China; 3 Office of Cancer Clinical Proteomics Research, National Cancer Institute, National Institutes of Health, Rockville, MD 20850, USA; 4 Department of Otolaryngology, Eye & ENT Hospital, Fudan University, Shanghai 200031, China

**Keywords:** Proteogenomics, Targeted cancer therapy, Integrated omics analysis

## Abstract

Advancements in molecular characterization technologies have accelerated targeted cancer therapy research at unprecedented resolution and dimensionality. Integrating comprehensive multi-omic molecular profiling of a tumor, proteogenomics, marks a transformative milestone for preclinical cancer research. In this paper, we initially provided an overview of proteogenomics in cancer research, spanning genomics, transcriptomics, and proteomics. Subsequently, the applications were introduced and examined from different perspectives, including but not limited to genetic alterations, molecular quantifications, single-cell patterns, different post-translational modification levels, subtype signatures, and immune landscape. We also paid attention to the combined multi-omics data analysis and pan-cancer analysis. This paper highlights the crucial role of proteogenomics in preclinical targeted cancer therapy research, including but not limited to elucidating the mechanisms of tumorigenesis, discovering effective therapeutic targets and promising biomarkers, and developing subtype-specific therapies.

## INTRODUCTION

Cancer remains a significant health problem worldwide. It is imperative to study tumorigenesis and improve cancer therapy. Traditional drug research is costly, tedious, time-consuming, and fraught with uncertainty. The next-generation sequencing (NGS) has revolutionized cancer therapy research (Wishart 2022). Since then, many cancer therapy strategies have been “rationally designed”, meaning that the target is first identified and then strategies developed. Successful examples include using BCR-ABL- and c-kit-targeting agents on chronic myeloid leukemia (Gonzalez-Fierro and Dueñas-González 2021). However, these strategies provide only limited treatment outcomes in clinical results. Gene mutations alone do not consistently predict prognosis or the response to therapies (Gerlinger *et al.* 2012; Simon and Roychowdhury 2013).

Proteogenomics is an interdisciplinary field that integrates proteomics and genomics, enabling researchers to incorporate genetic information into the interpretation of protein-level data. Proteogenomics was first introduced in 2004 and was initially applied to improve the annotation of genomes. Then the term has been comprehensively mapped to different aspects of biological activity, primarily focusing on genomes, transcriptomes, and proteomes (Jaffe *et al.* 2004; Tsang and Wong 2021). Early proteogenomic development was limited by proteomic technology. However, over the past few decades, advancements in proteomics, particularly liquid chromatography coupled with tandem mass spectrometry (LC-MS/MS), have been rapidly developed. One of the key advantages of proteogenomics is that proteogenomics provides a comprehensive and multi-dimensional approach that helps unravel the complexities of cancer by bridging the gap between genotype and phenotype, offering a holistic understanding of the cancer (Menyhart and Gyorffy 2021; Neagu *et al.* 2023; Zhang *et al.* 2020a).

A typical proteogenomic study involves multiple stages, ranging from sample collection to data analysis and interpretation. The main processes include: (1) Sample collection and preparation, which involves extracting nucleic acids (DNA and RNA) and proteins from biological samples. (2) Genomics and transcriptomics data generation, through DNA and RNA sequencing. (3) Proteomics data generation, primarily using LC-MS/MS analysis. (4) Data processing: raw sequencing and MS data are preprocessed (*e*.*g*., quality control, normalization, filtering) to reduce noise and enhance signal clarity. The genomic and transcriptomic data undergo variant calling and functional annotations. (5) Statistical analysis and discovery. The combined data are interpreted to identify key biological mechanisms, such as mutated genes that lead to aberrant protein expression or altered cellular pathways that drive cancer progression. (6) Validation and clinical application. Findings from the proteogenomic analysis (such as new biomarkers or drug targets) are validated through *in vitro* or *in vivo* experiments. The results are strongly recommended for inclusion in clinical assays or therapies, paving the way for precision medicine applications.

**Figure 1:**
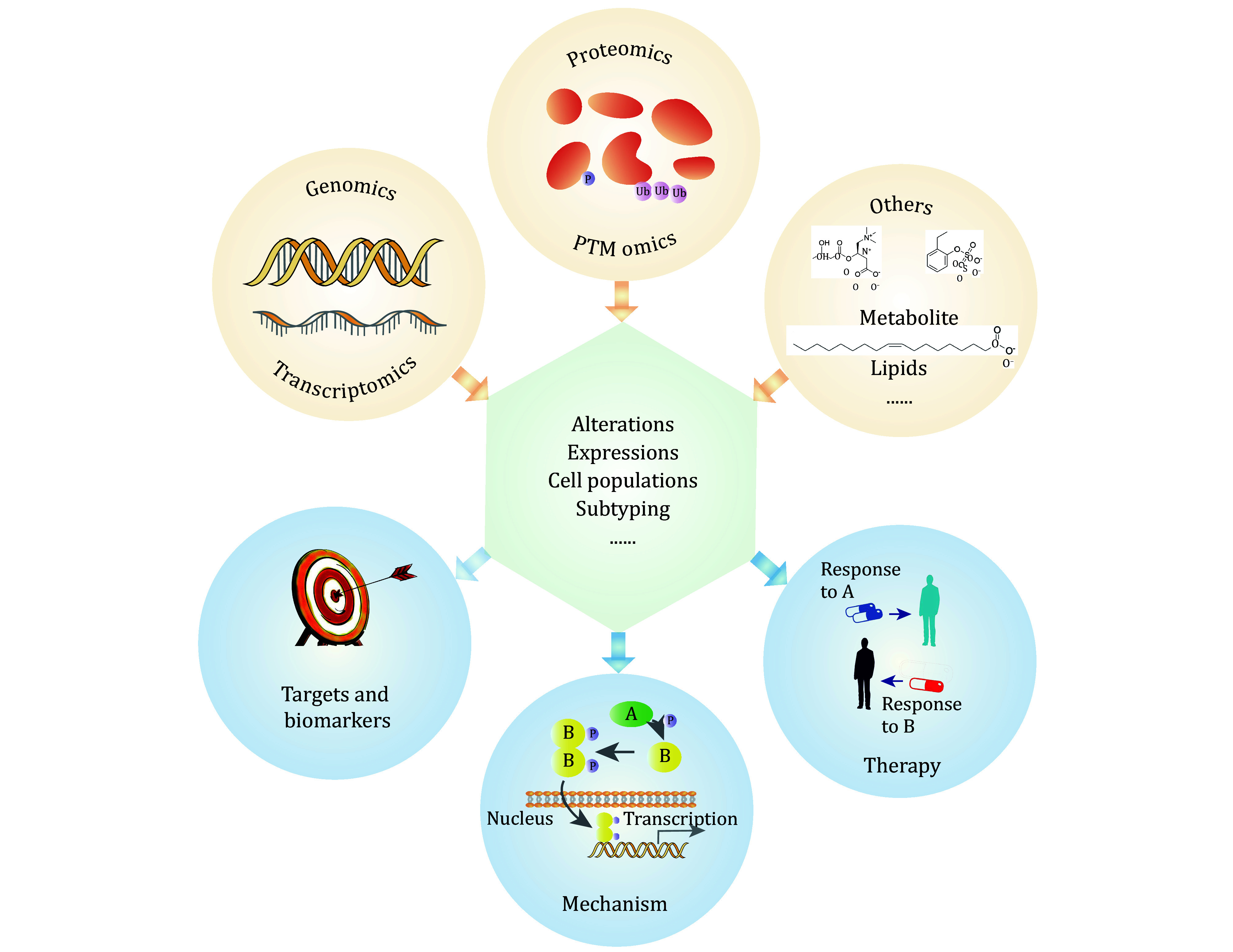
Proteogenomics and their applications in target cancer therapy research. The application of proteogenomics (mainly including genomics, transcriptomics, and proteomics) has become an indispensable tool in the diagnosis and treatment of various types of cancer. Through the assessment of alterations, molecular expressions, cell populations, sample subtyping, and other aspects, proteogenomics can provide invaluable insights into preclinical target cancer therapy research. This includes unraveling the mechanism of tumorigenesis, identifying potential targets and specific biomarkers, and predicting subtype-specific therapy

In this review, we first briefly introduce proteogenomics, including genomics, transcriptomics, proteomics, and post-translational modifications (PTM) omics, and then explore its various applications for preclinical cancer research. These data resources offer novel insights into tumors from various perspectives, further advancing personalized targeted cancer therapies (Fig. 1). We further introduce the multi-omics data analysis and pan-cancer analysis, which are popular strategies nowadays, emphasizing the role of proteogenomics in preclinical targeted cancer therapy.

## OVERVIEW OF PROTEOGENOMICS

Advancements in technology have expanded the scope of proteogenomics to encompass various areas (Wang *et al.* 2023). Still, the typical proteogenomic data include genomic, transcriptomic, and MS-based proteomics data.

### Genomics and transcriptomics

Genomics focuses on studying entire genomes. Transcriptomics examines RNA levels genome-wide, including qualitatively (identification of present transcripts, splice sites, and RNA editing sites) and quantitatively (detecting the expression of each transcript) (Hasin *et al.* 2017). Large-scale genome sequencing to characterize various types of cancers began in earnest as early as 2005. The most typical project is The Cancer Genome Atlas (TCGA) (Tomczak *et al.* 2015). Since 2013, The Cancer Genome Project has compiled approximately five million somatic alterations for over 7000 primary cancers (Alexandrov *et al.* 2013).

It has become increasingly feasible and cost-effective to perform genomics and transcriptomics studies (Bustamante *et al.* 2011). NGS enables high-throughput sequencing of DNA and RNA fragments (Kamps *et al.* 2017). The commonly used methods in proteogenomic studies for DNA sequencing are whole genome sequencing (WGS) and whole exome sequencing (WES). WGS can sequence the total DNA content and WES means sequencing of all protein-coding regions (*i*.*e*., the exome). The context and complexity of alterations such as point mutations, insertion-deletion mutations, and copy number variations (CNVs) can be identified (Ang *et al.* 2019; Petersen *et al.* 2017). The widely used method for transcriptomics is RNA sequencing (RNA-seq). It measures the total RNA from cells within bulk tissues and organs, providing an average expression level with mature technology and high throughput.

Conventional cancer omics research can only produce the average profile of cancer, regardless of heterogeneous cell types. Nowadays, single-cell omics have become popular for characterizing cells across different molecular levels. It enables the elucidation of cellular mechanisms and the deciphering of cell heterogeneity and diversity (Sun *et al.* 2024a). There are numerous single-cell omics technologies, among which single-cell transcriptomics has developed relatively quickly and paired with other omics (Baysoy *et al.* 2023; Bian *et al.* 2018; Wang *et al.* 2022). The popular tactics include genome-wide single-cell RNA sequencing (scRNA-seq) and single-nucleus RNA-Seq (snRNA-Seq) (Luecken and Theis 2019; Slyper *et al.* 2020; Sun *et al.* 2021). By uncovering cellular heterogeneity and building cell atlas resources, our understanding of the mechanisms of oncology can be improved.

### Proteomics and post-translational modifications omics

Proteins serve as the fundamental building blocks of life, driving essential biological processes. The vast majority of signal transduction pathways are governed by dynamic protein PTMs, which include phosphorylation, acetylation, glycosylation, *etc*. These processes contribute to regulating cellular biological signaling, providing a wealth of potential targets for cancer diagnosis and therapeutic strategies. Genomics and transcriptomics are unable to capture phenomena such as protein accumulation or degradation and the intricacies of PTM processes (Doll *et al.* 2019).

Proteomics has developed methods to directly measure and annotate protein abundance, interaction, localization, and modification states, offering new insights into tumor pathophysiology (Meissner *et al.* 2022; Suo *et al.* 2024; Xiao *et al.* 2020). There are several powerful tools for accurately assessing protein, including protein arrays (Akbani *et al.* 2014; Coarfa *et al.* 2021) and LC-MS/MS (Meissner *et al.* 2022). The latter method stands out as the most common method. Proteins are digested into peptides and sent to an LC instrument, which allows for the separation of complex peptide mixtures. The peptides are then ionized by the MS instrument, and the ions are manipulated using electromagnetic fields to measure their mass-to-charge (*m*/*z*) ratio. Quantitative proteomics focuses on measuring the abundance of proteins within a biological sample, encompassing both label-based and label-free methodologies (Meissner *et al.* 2022). The tandem mass tags (TMT) method and label-free quantification based on data-independent acquisition are of special concern (Ludwig *et al.* 2018; Zhang *et al.* 2020b).

PTMs refer to the chemical changes occurring at protein terminuses or amino acid side chains (Pan and Chen 2022). To identify these modified peptides, matrices with appropriate physical properties (such as charge and hydrophobicity) or antibodies are widely used for enrichment before LC-MS/MS detection (Silva *et al.* 2013). Take phosphoproteomics and glycoproteomics as examples. Phosphorylation is the most common type of PTM and is frequently studied (Silva *et al.* 2013). The classic enrichment strategies include immobilized metal affinity chromatography (IMAC) and metal oxide affinity chromatography (MOAC) (Xie *et al.* 2022). Glycobiology has garnered increasing attention for its significant role in tumorigenesis (Pan and Chen 2022). Yet glycosylation is a complex process and the intricate structure of glycoproteins constitutes a major technical difficulty. Some technologies, such as enzymes and probes, have facilitated the detection of glycans and glycosylation enzymes (Luo *et al.* 2023; Narimatsu *et al.* 2018; Tian *et al.* 2022).

The single-cell proteomic methods are still limited (Dwivedi *et al.* 2019). Before the availability of single-cell proteomics by mass spectrometry (SCP), most single-cell proteomics methods were antibody-based. For example, CyTOF (Cytometry by time-of-flight) utilizes rare metal isotopes for antibody labeling and can quantify labeled targets of single cells (Bandura *et al.* 2009; Li *et al.* 2018). SCP aims to identify and quantify the proteome of single cells using LC-MS/MS without antibodies. Since 2018, there has been an increasing boom of customized sample preparation platforms using SCP (Brunner *et al.* 2021; Tan *et al.* 2024). Nowadays, SCP requires further optimization in aspects such as instrumentation, sample preparation, and MS methods.

## ANALYSIS STRATEGIES AND PROMISING APPLICATIONS OF PROTEOGENOMICS IN PRECLINICAL TARGET CANCER THERAPY RESEARCH

**Figure 2:**
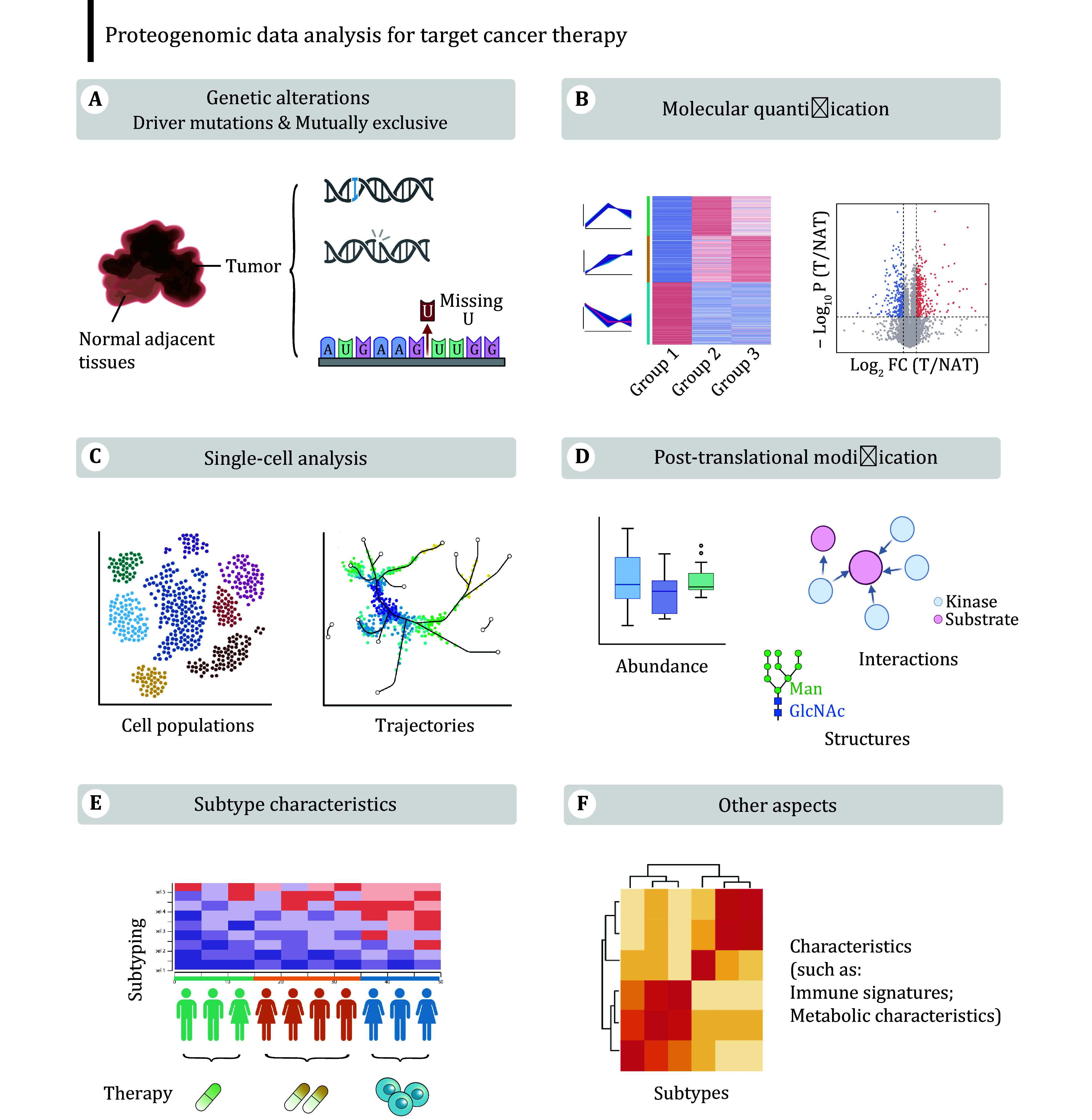
The proteogenomic data offers different perspectives for target cancer therapy research. Proteogenomics data provide comprehensive insights into various cancer samples (such as tumors, normal tissues, metastatic tissues, *etc*.), revealing their molecular biases and providing the basis for clinical precision medicine. The popular analytical aspects include, but are not limited to, identifying the genetic alterations and single-cell populations in tumor samples, comparing the RNA and protein expression levels among different kinds of samples, depicting the PTM patterns, clustering patients into distinct subtypes, analyzing immune landscapes, *etc*.

To date, most cancer proteogenomic studies have been exploratory, hypothesis-generating, so-called ‘landscape’ studies, aiming at producing the omics dataset (*i*.*e*. resource) of representative samples. Leveraging these datasets, proteogenomic applications primarily delve into fascinating aspects such as genetic alterations, differential expression, single-cell information, patterns of PTMs, subtype characteristics, and immune landscape (Fig. 2). Although the following descriptions are separated, they are often interactive during the analysis.

### Depiction of cancer proteogenomic landscape as valuable resources for subsequent exploration

The Clinical Proteomic Tumor Analysis Consortium (CPTAC) (Ellis *et al.* 2013) was established in 2011, which has developed a series of collaborating groups, to achieve proteomic analysis of different cancers and store large amounts of proteogenomic data. In order to remove the multiple sources of variability, CPTAC provides a standard and uniform proteomics analysis platform for all of the collaborators, including experimental design, MS methods, and database searching, making it possible to compare the data between different samples, cancer types, and institutions (Rudnick *et al.* 2016).

In proteogenomic studies, the selection of clinical samples is the crucial step. Tumors and their corresponding normal adjacent tissues (NATs) are the preferred samples, which directly represent the characteristics of the tumors. Biofluids are also highly sought after because they contain biomarkers that can help diagnose and monitor various medical conditions. WGS, WES, RNA-seq, miRNA-seq, scRNA-seq, proteomics, and PTM omics analysis are widely performed. These techniques provide insights into genetic and transcript alterations, RNA and protein quantification, levels of PTMs, and other molecular characteristics (Cao *et al.* 2021; Clark *et al.* 2019; Vasaikar *et al.* 2019; Wang *et al.* 2021a). Clinical information is also very important, especially survival information. By examining survival differences across different groups, researchers can determine whether the factor is key to clinical relevance (Gao *et al.* 2019; Liu *et al.* 2024). Patient characteristics such as gender, race, age, and tumor grade, are also documented.

Researchers have conducted comprehensive profiling across various cancer types over the past decade (Cao *et al.* 2021; Clark *et al.* 2019; Gao *et al.* 2019; Huang *et al.* 2021; Jayavelu *et al.* 2022; Kramer *et al.* 2022; Krug *et al.* 2020; Lehtio *et al.* 2021; Li *et al.* 2023b; Liu *et al.* 2024; Vasaikar *et al.* 2019; Wang *et al.* 2021a; Xiao *et al.* 2022). As data production increases, there are numerous public repositories besides TCGA and CPTAC (Clark and Lillard 2024; Subramanian *et al.* 2020). Multi-omics data provide the landscape of cancer characterization fromssss various dimensions and perspectives, providing a data foundation for cancer research.

Identification of genetic and transcript alterations for uncovering the mechanisms of tumorigenesis and new therapeutic targets

Among the numerous alterations observed, only a select few — known as driver mutations — have the potential to initiate and propel cancer progression (Ostroverkhova *et al.* 2023). They are associated with clinical phenotypes and different risk factors, and some of them can be regarded as therapeutic targets and biomarkers (Cancer Genome Atlas Research 2012; Schulze *et al.* 2015). Since the 1970s, the list of driver genes has been growing gradually, and there are now more than 500 cancer genes (Martínez-Jiménez *et al.* 2020). Somatic changes in cancer genomes often result in transcript alterations. Various forms of RNA alterations, such as altered splicing and gene fusions, have been described in cancer (Calabrese *et al.* 2020).

By identifying the genomic drivers, we can enhance our understanding of tumorigenesis and identify potential therapy targets. One group applied exome sequencing to hepatocellular carcinoma (HCC), identifying mutational signatures associated with distinct risk factors. They found that 28% of patients harbored at least one alteration potentially targeted by an FDA-approved drug, which was potential treatment chances (Schulze *et al.* 2015). Another group carried out a proteogenomic analysis of HCC across different clinical stages and etiologies. Authors identified the driver gene list, including UBQLN4 and RRB2M, and suggested them as therapeutic targets (Ng *et al.* 2022). Alterations not only can be directly inhibited by targeted drugs but also might indirectly influence drug sensitivity, such as the relevance between NQO1 expression and HSP90 inhibitors (Schulze *et al.* 2015).

A series of filtering criteria can be used to identify putative key drivers (Cao *et al.* 2021; Huang *et al.* 2021; Vasaikar *et al.* 2019). For instance, Vasaikar *et al*. performed a systematic proteogenomic analysis of colon cancer and prioritized genetic alterations. By correlating copy number variations with mRNA and protein levels, and examining the consistent differences in protein expression between paired tumor and NAT samples, they prioritized 90 somatic CNV genes for further investigation. These genes and significantly mutated genes are enriched in cell proliferation, cell death, and Hippo signaling pathways (Vasaikar *et al.* 2019).

Many proteogenomic studies have observed mutually exclusive genomic alterations (Chen *et al.* 2020; Gao *et al.* 2019; Gillette *et al.* 2020; Huang *et al.* 2021). Two plausible situations may explain the mutually exclusive alterations. The first point is that alterations are functionally equivalent, meaning changes to other genes offer no additional benefit (Ciriello *et al.* 2012). Another is synthetic lethality (SL) interactions, where the combination of two genetic abnormalities leads to lethality, despite each being harmless on its own (Ciriello *et al.* 2012; Lee *et al.* 2021). One of its most significant applications is in the formulation of drug combination strategies. Zhang *et al*. developed the Synthetic Lethality Knowledge Graph (SLKG) to explore the SL and synthetic dosage lethality. They integrated genomics and drug datasets into a drugs-targets-indications knowledge graph. A computational model identified potential drug combinations, with the top-ranked SL gene pair CDKN2A + RRM2 validated through experimental methods, indicating that the RRM2 inhibitor may be a promising treatment for melanoma patients with CDKN2A mutations (Zhang *et al.* 2021a).

### Quantification of mRNA and protein for revealing the cancer mechanisms and potential biomarkers

The levels of mRNA and proteins are extensively analyzed to detect gene expression levels and signaling pathway activity among different samples (such as comparison between tumors and NATs), thereby offering a distinct molecular foundation. Compiling lists of differentially expressed genes along with their enrichment analysis can highlight potential key factors in tumorigenesis and uncover mechanisms associated with cancer development (Li *et al.* 2023c; Xu *et al.*^2020^).

In recent years, the combination of bulk RNA-seq and scRNA-seq analysis has been a popular strategy, offering diverse and complementary perspectives on biological research (Bao *et al.* 2021; Bischoff *et al.* 2021; Briggs *et al.* 2021; Liu *et al.* 2022a, b; Sun *et al.* 2024b). In prostate cancer studies, through mRNA expression analysis at both bulk and single-cell levels, researchers have identified the JAK/STAT signaling pathway as a crucial factor in lineage plasticity, providing a theoretical basis for therapeutic targets (Chan *et al.* 2022; Deng *et al.* 2022). Loss of function of TP53/RB1 and activation of SOX2 occur in lineage plasticity-driven resistance in metastatic castration-resistant prostate cancer (mCRPC). Deng *et al*. analyzed transcriptomic changes induced by TP53/RB1 deficiency and overexpression of SOX2 in cell lines. They found that JAK/STAT pathway was the only cancer-related pathway that was concomitantly altered with the dysfunction in mCRPC. Two major clusters of PCa cell subpopulations were identified by scRNA-seq, with increased expression of JAK/STAT signaling genes observed in the TP53/RB1-low subpopulation. These results indicated that JAK/STAT members may serve as promising targets for resensitizing resistant tumors to AR-targeted therapy (Deng *et al.* 2022).

It’s worth noting that the weak correlation between mRNA and proteins suggests the presence of multiple post-transcriptional modifications (Chakraborty *et al.* 2018; Clark *et al.* 2019; Gao *et al.* 2019), underscoring the significance of quantifying proteins directly in biomedical research. Clear cell renal cell carcinoma (CCRCC) is classified as a metabolic disease (Wettersten *et al.* 2017). A study revealed a prominent inconsistency of oxidative phosphorylation (OXPHOS) mRNA and protein expression in CCRCC tumors. Proteins and mRNAs in the glycolysis pathway were upregulated, and the mRNA expression level of the OXPHOS components was not changed. However, proteins associated with OXPHOS were significantly downregulated, reflecting the regulation of OXPHOS primarily at the translational level (Clark *et al.* 2019).

The differences in molecular expression levels among different samples can be analyzed in conjunction with clinical characteristics. For example, smoking is an important factor contributing to lung cancer. One group defined a smoking score (SS) to study the relevant phenotypes in lung adenocarcinoma (LUAD). Pathways such as cell cycle and signaling by FGFR were reduced, while P53 pathway and apoptosis were higher in NATs with high SS, but these patterns were reversed in tumors. Notably, the abundance of ARHGEF5 and its phosphosite Y1370y were elevated in tumors from strict never-smokers, highlighting a potential oncogenic mechanism in never-smokers (Gillette *et al*. 2020).

### Single-cell omics capturing tumoral and microenvironmental heterogeneity

The core application of single-cell omics is defining cell populations, shedding light on how specific cell types contribute to tumor characteristics. The application of single-cell omics to cell biology and preclinical research is still in its infancy, yet it holds significant potential for elucidating the cellular landscape (Baysoy *et al.* 2023). The tumor microenvironment (TME) is composed of various cellular components, which could provide growth and anti-apoptotic benefits to surviving cancer cells, decrease drug penetration, and alter disease characteristics while distorting clinical outcomes (Sun 2016). Single-cell omics has revolutionized our understanding of the TME, allowing for the exploration of the notable differences in its cell types.

Understanding the complex disturbance within the TME is crucial for understanding oncology (Cao *et al.* 2023; Kim *et al.* 2024; Luo *et al.* 2024; Ramberger *et al.* 2024; Sorin *et al.* 2023; Wu *et al.* 2023). Take renal cell carcinoma (RCC) as an example. Metastatic CCRCC is considered an immunotherapy sensitive tumor while others are not (Zhang *et al.* 2021c). There are some studies that reveal the immune-related heterogeneity of RCC through single-cell RNA-seq (Li *et al.* 2023b, 2024; Zhang *et al.* 2021c). Zhang *et al*. performed scRNA-seq on patients of RCCs and benign adjacent tissues. High immune cell infiltration was observed in CCRCC compared with classic chromophobe RCC, which is consistent with their responses to immunotherapy. They identified a list of genes that were associated with clinical tyrosine kinase inhibitors followed by nivolumab outcomes. It is notable that genes with a negative correlation with the response were primarily expressed in the major endothelial cluster in CCRCC. This indicated that metastatic CCRCC patients with high endothelial cell levels may respond poorly to immunotherapy (Zhang *et al.* 2021c).

Single-cell sequencing can model dynamic cellular changes by analyzing expression pattern similarities to construct differentiation and growth trajectories. Comparing characteristics of different trajectories offers insights into cancer development and therapy response (Trapnell *et al.* 2014; Van den Berge *et al.* 2020). Prostate cancer (PCa) is an extensive heterogeneous cancer, with androgen-deprivation therapy (ADT) being the standard treatment for metastatic cases. Bian *et al*. performed a single-cell analysis of PCa tissues and clustered myeloid cells into seven cell types. They found that IL1B-NLRP3 macrophages were enriched in ADT-treated patients. Pseudotime analysis identified two evolutionary trajectories of macrophages. Key genes defining these branches were identified, suggesting that fate2, primarily composed of IL1B-NLRP3 subsets, represents a pro-inflammatory phenotype or immune activation state. These results indicated that ADT might promote the formation of IL1B-NLRP3 macrophages, which may have anticancer effects (Bian *et al.* 2024). Another research of PCa revealed FOXA2 as a neuroendocrine lineage-pioneering transcription factor by inferencing cell differentiation progression trajectory. Further studies indicate that FOXA2 can drive KIT pathway activation in neuroendocrine cells in PCa (Han *et al.* 2022).

### Annotation of PTMs for uncovering molecular mechanisms and potential therapeutic targets and biomarker

PTMs play a crucial role in enhancing the functional diversity of proteins and providing a complex layer of regulation essential for cellular processes. Analyzing trends in PTM site expression helps researchers explore tumor mechanisms at the post-translational level. For example, Vasaikar *et al*. discovered that RB1 was amplified and protein was upregulated in colorectal cancer (CRC). This phenomenon contradicted the role of RB1 as a tumor suppressor gene (TSG). Then they explored the phosphoproteomic data and found that RB1 phosphorylation is upregulated. Through their analysis, they concluded that RB1 phosphorylation, rather than RB1 mutations, drives tumor growth and has an antiapoptotic effect (Vasaikar *et al.* 2019).

Kinases are one of the most intensively pursued targets in antitumor drug research, due to their critical roles in cellular signaling (Wu *et al.* 2015). There is considerable interest in measuring phosphorylation abundance levels and kinase activity to identify potential therapeutic targets (Huang *et al.* 2017; Krug *et al.* 2020). Liu *et al*. subtyped a cohort of small cell lung cancer (SCLC) into four clusters. By comparing the difference in phosphosite abundance among different samples, they identified a subtype characterized by elevated receptor tyrosine kinase (RTK) signaling and suggested RTK inhibitors as an effective strategy for these tumors (Liu *et al.* 2024). There has also been increased focus on inferring kinase activity, understanding kinase-substrate networks, and assessing their implications for drug responses (Savage and Zhang 2020). Several algorithms are helping to predict the kinase activity such as KSEA (kinase-substrate enrichment analysis) (Wiredja *et al.* 2017), IKAP (Inference of kinase activities from phosphoproteomics) (Mischnik *et al.* 2016), *etc*. (Piersma *et al.* 2024). McDermott *et al*. performed a proteogenomic study on ovarian high-grade serous cancer (HGSC). Using KSEA revealed significantly increased activity of multiple kinases associated with cell-cycle control in tumors, including CDKs and AURKA. These results suggest the possible therapeutic target for HGSC and prompted an investigation of mechanisms related to cell cycle regulation and replication stress in this tumor (McDermott *et al.* 2020).

Glycobiology has become increasingly prominent in cancer research, involving numerous vital biological processes such as inflammation, cell–cell adhesion, and cell–matrix interaction (Pinho and Reis 2015). Aberrant glycans and glycosylation enzymes have been identified as potential targets for cancer diagnosis and treatment (Pinho and Reis 2015; Tian *et al.* 2022). For example, Rolland *et al*. generated the landscape of N-glycoproteins from lymphoma tissue biopsies and cell lines. They focused on anaplastic lymphoma kinase-positive anaplastic large cell lymphoma (ALKALCL) and several proteins with cytokine receptor activity. Among them, IL-31Rβ was exclusively expressed in ALKALCL cell lines and regulated by ALK. Through functional verification by biological experiments such as RNA interference, IL-31Rβ is considered a therapeutic target for ALKALCL (Rolland *et al.* 2017).

### Patient stratification for subgroup-specific therapeutics

Different pathogenic factors can activate diverse molecular mechanisms, leading to significant heterogeneity among patients. A popular strategy is classifying clinical samples and analyzing their distinct characteristics. Common methods for classifying include consensus clustering (Clark *et al.* 2019; Monti *et al.* 2003) and non-negative matrix factorization (NMF) (Cao *et al.* 2021; Huang *et al.* 2021; Rappoport and Shamir 2018). By patient stratification, precise treatment plans for different subtypes can be formulated, aiding the selection of more effective treatments for individuals (Laurent-Puig *et al.* 2001; Menyhart and Gyorffy 2021; Nguyen *et al.* 2020; Smith *et al.* 2022).

Patients in different subtypes can exhibit significant differences in various characteristics, such as mutations, RNA or protein or PTM site abundance levels, and pathway activity. Through subtyping of patient cohorts, potential biomarkers or targets can be identified, aiding the selection of more effective treatments for individuals. Triple-negative breast cancer (TNBC) is known for its heterogeneity and treatment difficulties (Burstein *et al.* 2015; Gong *et al.* 2022; Stewart *et al.* 2020; Xiao *et al.* 2022). Burstein *et al*. carried out proteogenomic characterization on 198 TNBC tumors, confirming four distinct subtypes based on RNA expression profiling. They analyzed subtype specific characterization and identified subtype-specific targets. One of the subtypes exhibited activation of immunoregulation pathways and STAT mediated pathways, indicating that STAT inhibitors and cytokine/cytokine receptor antibodies might be suitable for this subtype (Burstein *et al.* 2015). Another study on TNBC in China divided another cohort into four subtypes and found that the JAK/STAT3 signaling pathway was noticed in the mesenchymal-like subtype, suggesting that STAT3 inhibitors could be a promising treatment strategy for this subgroup (Jiang *et al.* 2019).

Subtypes identified using different datasets may show discrepancies in their results. Several studies highlight the advantages of proteomic typing, such as providing a more accurate indication of clinical responses and patterns (Gao *et al.* 2019; Macklin *et al.* 2020; Xu *et al.* 2020). Gao *et al*. performed clustering of HCC based on differentially expressed proteins between tumor and NAT, and established several screening criteria to identify representative proteins for subtypes. They pinpointed PYCR2 and ADH1A exhibited significant differential expression across the proteomics subtypes and strongly correlated with patient survival. These proteins were identified as prognostic biomarkers with potential clinical utility (Gao *et al.* 2019). The same team conducted another research for intrahepatic cholangiocarcinoma and identified four proteomic subgroups (S1–S4) with divergent genomic and clinical features. They discovered that subtypes S1 and S2 exhibited greater sensitivity to EGFR inhibitors, while cell lines other than those in S1 and S3 demonstrated sensitivity to Gemcitabine and Paclitaxel (Dong *et al.* 2022).

### Analysis of the immune landscape suggesting the applicability of immunotherapy

Immunotherapy is one of the important methods of cancer therapy, and proteogenomic analysis aids in the research of immunotherapy from many aspects. The composition and density of immune cells in the TME, which significantly impact tumor progression and the effectiveness of anti-cancer treatments, can be deconstructed by various methods (Sturm *et al.* 2019). Besides, tumor-specific antigens can stimulate T cell-mediated antitumor immune responses and serve as available targets for cancer immunotherapy.

Based on bulk proteomic and gene expression data, there are many tools such as xCell (Aran *et al.* 2017) and BayesDeBulk (Petralia *et al.* 2023), that can analyze and classify the immune and stromal cell composition (Sturm *et al.* 2019). Tumors can be clustered based on cell signatures, such as being grouped into immune hot, warm, and cold clusters, allowing for the investigation of disparities among these groups, and providing insights into cancer immunotherapy (Cao *et al.* 2021; Gillette *et al.* 2020; Krug *et al.* 2020; Liu *et al.* 2024; Satpathy *et al.* 2021; Wang *et al.* 2021a). Pancreatic ductal adenocarcinoma (PDAC) is a highly heterogeneous and aggressive tumor, usually resistant to immune checkpoint inhibitors. One group of CPTAC profiled the proteogenomic characterization of PDAC and delineated the cellular composition of tumors by xCell. They classified the tumors into four clusters according to the cell composition and characterized the ‘immune-cold’ phenotype. These tumors showed reduced expression of endothelial adhesion proteins and increased activity of VEGF and hypoxia pathways, which were important to the remodeling of endothelial cells. Immune-cold samples also exhibited elevated levels of glycolysis. Researchers proposed that targeting glycolysis and endothelial cell remodeling, could be therapeutically leveraged to enhance antitumor immunity in immune-cold PDACs, which can be verified by clinical outcomes (Cao *et al.* 2021).

Single-cell omics data provide a more intuitive representation of the cellular composition within the TME (Bärthel *et al.* 2023; Cao *et al.* 2023; Christodoulou and Zaravinos 2023; Chung *et al.* 2017; Gessain *et al.* 2024). Zhang *et al*. carried out single-cell analysis for TNBC patients. A total of 22 patients were included in this cohort, with half receiving paclitaxel monotherapy and the other half receiving paclitaxel in combination with atezolizumab. Through measuring the correlations between cell proportion change with tumor size changes, T cells notably exhibited a positive role in PD-L1 blockade therapy. Researchers identified two T cell clusters (*i*.*e*., CD8-CXCL13 and CD4-CXCL13), which showed decreased levels in paclitaxel treatment responders but increased levels in responders following paclitaxel plus atezolizumab treatment. These results demonstrated the important role of CXCL13+ T cells in predicting the clinical outcomes of anti-PD-L1 therapy (Zhang *et al.* 2021b).

Neoepitopes are identified by the adaptive immune system and act as a mechanism for the immune system to distinguish cancer cells from normal cells, serving as the promising target. Somatic or passenger mutations in the tumor lead to the formation of new epitopes or neoepitopes (Brennick *et al.* 2017; Ren *et al.* 2024). MS is the primary technique for directly identifying neo-epitopes displayed on tumor cells (Xiang *et al.* 2022). Dong *et al*. performed proteogenomic analysis for intrahepatic cholangiocarcinoma (iCCA). FGFR2 fusions were found in 11.9% (30/253) in this cohort and the common fusion partners included BICC1. For the two most common FGFR2::BICC1 fusion proteins, a set of fusion-derived peptides was built. Although the results showed a low frequency of neoepitope T cell reactivity, some peptides derived from FGFR2 fusion proteins exhibited strong immunogenicity and were potential targets for immune antigens (Dong *et al.* 2022).

## INTEGRATIVE ANALYSIS

### Combined analysis of omic data

Comprehensive proteogenomic studies treat the tumor as a system, offering insights from myriad layers, thereby promising a more systematic and holistic understanding of the intricate nature of cancer and drug interactions. Therefore, integrating proteogenomic data enhances the credibility and reliability of research outcomes, ultimately refining preventive measures and treatment strategies for cancer (Subramanian *et al.* 2020; Zhang *et al.* 2020a).

There are some common strategies to implement integrated analyses. The first strategy is data mapping, where genomic features are mapped to transcriptomic and proteomic data to identify relationships between genetic variations and their downstream effects. A specific CNV or mutation could be linked to gene expression changes, which then influence protein abundance or PTM levels. The second is comparing data from different omics based on shared identifiers (*e*.*g*., gene symbol). Statistical methods (*e*.*g*., statistical tests and regression) can be used to identify relationships between multiple datasets, such as gene expression, protein abundance and PTM abundance data. The third strategy is the application of machine learning methods. For example, unsupervised learning is widely used for patient subtyping. Clustering algorithms (*e*.*g*., k-means, hierarchical clustering, t-SNE) are used to identify patterns across multiple omics data types without predefined labels. Additionally, there are many tools and software available for multi-omics integration.

Combining the different levels of omics data can provide novel and comprehensive insights into cancer pathogenesis, further suggesting the identification of candidate biomarkers and therapeutic targets. A typical example was provided by Satpathy *et al*. They studied lung squamous cell carcinoma samples and found that NSD3 is an amplified cancer-related gene within a focal amplicon that includes FGFR1. Their proteomics data showed that NSD3, not FGFR1, was significantly upregulated in tumors with high FGFR1 amplification. These results suggest the limited effectiveness of FGFR1-targeted therapies and highlight NSD3 as a potential alternative treatment (Satpathy *et al.* 2021). Gillette *et al*. focused on therapeutic vulnerabilities in LUAD and integrated mutations and phosphosites data. They found that KRAS mutant tumors showed significant upregulation of SOS1 phosphorylation on S1161, while in EGFR mutant tumors was PTPN11/Shp2 at Y26. This may indicate new therapies and PTPN11/Shp2 inhibitors perform well in preclinical trials (Gillette *et al.* 2020).

There are numerous studies revealing that the median correlation between mRNA and protein abundance is weak (around 0.4), indicating post-transcriptional regulation. Further analysis indicates that the degree of correlation between mRNA and protein levels is related to the gene’s biological function. The weaker correlations were observed in highly stable and abundant proteins (*e*.*g*. ribosomes, spliceosomes), while proteins that are more dynamically regulated in response to disturbances (such as a metabolic process) show stronger correlations (Clark *et al*. 2019; Gao *et al*. 2019; Gillette *et al*. 2020; Zhang *et al*. 2016). These findings suggest that discrepancies between mRNA and protein data are common and normal, reflecting the regulation at different biological levels. To address these issues, we can analyze the data in conjunction with the protein’s function and select the appropriate data according to the specific research requirements.

Genetic variants can broadly impact the transcriptional, translational, and post-translational product. If the effect is on the expression of itself, it is referred to as ‘cis-effect’; if it affects other genes, it is termed ‘trans-effect’ (Clark *et al.* 2019; Li *et al.* 2023c). The correlation between RNA and CNV changes is usually more significant than that between protein and CNV changes (Gao *et al.* 2019; Gonçalves *et al.* 2017). Observing the dosage cascade regulation can provide novel insights into the mechanisms of cancer. A group conducted proteogenomic analysis on Chinese CCRCC patients. The data showed that the center of CNVs with cis-effect included 3q, where the copy number was negatively correlated with survival. The researchers prioritized genes located at 3p that showed significant cis-effects and evaluated their correlation with survival. They focused on the SLC4A7, which is crucial for cellular net acid extrusion and maintaining intracellular pH balance, indicating the significance of SLC4A7 in regulating cellular pH balance in tumors (Qu *et al.* 2022).

It is worth mentioning that a popular strategy is multi-omics clustering. The mutations, CNVs, mRNA expression, protein, and PTM site abundance can all be used as input features for data clustering (Krug *et al.* 2020; Wang *et al.* 2021a), providing the basis for subtype-specific therapeutic chances. Liu et al. subtype SCLC tumors into four nmf subtypes based on multi-omics data. The nmf1 subtype was characterized by high replication stress and neuroendocrine differentiation, suggesting the response to drugs that exacerbate genome instability; the nmf2 subtype showed a high abundance of DLL3, suggesting the response to anti-DLL3 therapies; the nmf3 subtype showed elevated RTK signaling, which may be response to RTK inhibitor treatment; the nmf4 subtype displayed high expression of MYC and POU2F3, indicating its benefit from AURK inhibitors (Liu *et al.* 2024).

There are also some designed statistical methods to integrate and analyze multiple types of omics data, such as Multi-Omics Factor Analysis (MOFA) (Argelaguet *et al.* 2018, 2020) and mixOmics (Rohart *et al.* 2017). MOFA infers a set of latent factors (LF) representing sources of variability from different omics data sets across the samples (Argelaguet *et al.* 2018). It helped researchers to reveal factors associated with clinically relevant biology (Herbst *et al.* 2022). Sharma *et al*. employed MOFA+ to integrate multi-omics data from breast cancer patients. LF1 and LF2 represented most of the heterogeneity showing a strong association with tumor grade. LF1 and LF13 were significantly associated with BC metastasis and death. Based on LF1, LF2, and LF13, patients were grouped into three clusters, providing a new basis for the characterization and understanding of BC (Sharma *et al.* 2024).

### Pan-cancer analysis

Various cancers exhibit unique characteristics. Yet, overarching commonalities exist across an array of cancers, such as significant driver mutations, altered pathways, and immune signatures. These similarities highlight the value of a strategy called ‘pan-cancer’, which targets traits shared among various cancer types (Chen *et al.* 2021). Pan-cancer studies focus on the molecular similarities and differences across various cancer types, rather than solely on the tissue of origin.

After completing genomic investigation across 33 cancer types, TCGA demonstrated the significance of the pan-cancer study and published the project namely the ‘Pan-Cancer Atlas project’ (Bailey *et al.* 2018). Latterly in 2020, the Pan-Cancer Analysis of Whole Genomes (PCAWG) consortium was established, integrating 2,658 whole-cancer genome data shared by compute clouds (Consortium 2020). The comprehensive cancer genome analysis studies were carried out from many aspects, such as driver mutations (Consortium 2020), non-coding somatic drivers (Rheinbay *et al.* 2020), mutational signatures (Alexandrov *et al.* 2020), *etc*. PCAWG mainly concentrates its attention on the analysis of genomics data. CPTAC consortium has also published a series of proteogenomic pan-cancer studies and provided a comprehensive public data resource, profiling many aspects of pan-cancer, including the characterization of oncogenic drivers, the pattern of PTMs, DNA methylation, tumor immunity, and therapeutic targets (Geffen *et al.* 2023; Li *et al.* 2023a, c; Liang *et al.* 2023; Petralia *et al.* 2024; Savage *et al.* 2024). They generated the harmonized proteogenomic and clinic data over ten cancer types and also established a pipeline for pan-cancer sample collection and data harmonization (Li *et al.* 2023a).

Pan-cancer studies can facilitate the identification of potential cancer drivers and prognostic relevance across different cancer types (Petralia *et al.* 2024; Wang *et al.* 2021b; Zack *et al.* 2013). Savage *et al*. published a pan-cancer study to expand the landscape of therapeutic targets. Through analyzing the associations between alteration of TSGs and variation in protein abundance, they focused on the TP53-TOP2A pairs in EC. TOP2A can be targeted by chemotherapy agents such as doxorubicin, whose abundance was significantly higher in tumors harboring TO53 loss. These results suggested that TP53 loss may serve as a biomarker for selecting EC patients who are suitable for treatment with doxorubicin (Savage *et al.* 2024). Li *et al*. provided insights into shared oncogenic drivers across ten types of cancer. They identified that PLOD2, UBE2C, and MARCKSL1 were commonly differentially expressed between NATs and tumors. Among these genes, PLOD2 abundance was associated with survival and can be regarded as a pan-cancer prognostic biomarker (Li *et al.* 2023c).

## SUMMARY AND PERSPECTIVES

Proteogenomic technologies are revolutionizing preclinical research by objectively and comprehensively delineating the entire molecular landscape. Proteogenomic datasets contain information from various biological dimensions, helping to uncover the mechanisms of tumorigenesis, identify potential therapeutic targets and specific biomarkers, and drive the development of precision medicine. The ultimate goal of preclinical proteogenomic studies is to create strategies for patient-specific cancer therapy, taking into account the heterogeneity of different cancers.

Artificial intelligence (AI) has been rapidly applied in preclinical cancer research because of its predictive power and precision. A major area of AI research is centered on enabling machines to derive general rules from data, a process known as machine learning (ML), to make predictions about previously unseen samples. ML algorithms can identify hidden patterns and relationships within large datasets from diverse sources, which might not be evident through traditional methods (Rehman *et al.* 2024). AI-driven methods can uncover novel targets that were previously unrecognized or ignored and develop more robust classification approaches for clinically relevant cancer subtypes (Boniolo *et al.* 2021). In the future, we look forward to AI, especially ML, playing a key role in large-scale data analysis, and further promoting the development of personalized therapies based on individual molecular profiles.

Despite significant progress, we still have a long way to go in fully understanding cancer and overcoming the various challenges. Firstly, advancements in proteomic technologies have not progressed as rapidly, especially for single-cell proteomic analysis (Olivier *et al.* 2019). There are numerous challenges such as detection sensitivity, dynamic range, analytical throughput, and equipment costs. It is worth mentioning that advanced protein sequencing techniques may provide more convenient and accessible platforms for proteomic research (Guo *et al.* 2024; Singh 2022). For example, nanopore technology can directly capture molecular nature as single molecules pass through nanopores, enabling the sequencing of intact protein (Motone *et al.* 2024). Secondly, a major challenge lies in integrating and interpreting the vast array of results from diverse sources. There are many public data resources and trying to combine data from different public resources is complicated. Different experimental and computational methods can yield inconsistent results, leading to potential false positives or negatives. Besides, there is difficulty trying to extract authentic knowledge out of large multi-omic data sets. With the impending of AI, machine learning and similar technologies may become especially valuable for interpreting large-scale data (Darbandi *et al.* 2024; Sun *et al.* 2022; Xiao *et al.* 2021). Thirdly, translating biological cancer research into clinical practice remains challenging. There are some reasons: (1) Tumor tissues exhibit significant heterogeneity, and samples used in proteogenomic studies can only represent a subset of tumor characteristics. (2) The patients represent only a subset of the population and do not reflect the entire patient population (Liu *et al.* 2024). Even so, studies have showcased the potential of current proteogenomic findings for clinical applications (Klomp *et al.* 2024; Liu *et al.* 2024).

Looking ahead, there are several future directions in proteogenomic research. Firstly, proteogenomics is expected to evolve into single-cell spatial multi-omics, three-dimensional spatial omics, and spatiotemporal multi-omics. These innovations will generate more detailed, high-resolution data, providing valuable insights into the dynamic alterations in tumor molecular structures throughout progression and treatment. Secondly, an increasing number of patients, including those from different regions, populations, and various cancer subtypes, should be included in research cohorts to enhance the generalizability and representativeness of the studies. Thirdly, diverse research perspectives are needed rather than a standardized, assembly-line approach to analysis, which may rely on the development of bioinformatics tools and AI. For instance, we should conduct clinically-driven analyses, focusing on specific clinical issues such as drug treatment, and tailor the analysis accordingly. Fourthly, the ultimate goal of proteogenomic research is to advance disease treatment. If the identified targets or biomarkers are used in clinical trials and yield positive results, the study will attract widespread attention. Collaboration among experts from diverse backgrounds becomes increasingly crucial (Alseekh *et al*. 2021; Doll *et al*. 2019), for developing improved treatment strategies for patients and advancing precision medicine.

## Conflict of interest

Yuying Suo, Yuanli Song, Yuqiu Wang, Qian Liu, Henry Rodriguez and Hu Zhou declare that they have no conflict of interest.
